# Survival outcomes of hepatectomy for stage B Hepatocellular carcinoma in the BCLC classification

**DOI:** 10.1186/s12957-017-1229-x

**Published:** 2017-08-22

**Authors:** Toshiya Kamiyama, Tatsuya Orimo, Kenji Wakayama, Shingo Shimada, Akihisa Nagatsu, Hideki Yokoo, Hirofumi Kamachi, Kenichiro Yamashita, Tsuyoshi Shimamura, Akinobu Taketomi

**Affiliations:** 1 Department of Gastroenterological Surgery I, Hokkaido University Graduate School of Medicine, North 15, West 7, Kita-ku, Sapporo, 060-8638 Japan; 2 Department of Transplantation Surgery, Hokkaido University Graduate School of Medicine, Sapporo, Japan; 3 Department of Organ Transplantation, Hokkaido University Hospital, Sapporo, Japan

**Keywords:** Hepatocellar carcinoma, Hepatectomy, BCLC staging.

## Abstract

**Background:**

Because hepatectomy is not recommended in patients with stage B hepatocellular carcinoma (HCC) of the Barcelona Clinic Liver Cancer (BCLC) staging, we evaluated the survival outcomes of hepatectomy for stage B in the BCLC system.

**Methods:**

Data were collected from 297 consecutive adult stage B patients who underwent curative hepatectomy for HCC between 1996 and 2014 in Hokkaido University Hospital. Overall survival (OS), disease-free survival (DFS), and risk factors were analyzed using the Kaplan–Meier method. Independent prognostic factors were evaluated using a Cox proportional hazards regression model. AP-factor (alpha-fetoprotein [AFP] × protein induced by vitamin K absence or antagonism factor II [PIVKA-II]) was categorized according to the serum concentrations of AFP and PIVKA-II: AP1 (AFP < 200 ng/ml and PIVKA-II < 100 mAU/ml), AP2 (AFP × PIVKA-II < 10^5^), and AP3 (AFP × PIVKA-II ≥ 10^5^).

**Results:**

There were 130 deaths among our 297 stage B patients (43.8%). The causes of death in these cases were HCC recurrence (*n* = 106; 81.5%), liver failure (*n* = 7; 5.4%), and other causes (*n* = 17; 16.1%). The operative mortality rate was 0.34% (1/297). The 5-year OS and DFS rates for the stage B cases were 54.3 and 21.9%, respectively. By multivariate analysis, tumor number and AP-factor were risk factors for both survival and recurrence that were tumor related and could be evaluated preoperatively. The study patients with stage B HCC were classified into three groups by tumor number (B1, 1; B23, 2 or 3; B4over: ≥4) and into three groups stratified by AP-factor (AP1, AP2, and AP3). The 5-year OS rates of B1, B23, and B4over were 63.6, 52.3, and 29.0%. The 5-year OS rates of AP1, AP2, and AP3 were 67.6, 65.2, and 39.1%. Stratified by the 5-year OS rate, stage B HCC patients were classified into three subgroups (A-C).The 5-year OS rates of groups A (B1 or B23 and AP-1 or AP-2), B (B1 or B23 and AP-3, or B4over and AP-1 or AP-2), and C (B4over and AP-3) were 69.5, 43.7, and 21.3%.

**Conclusion:**

Stage B HCC patients with a tumor number ≤ 3 and/or AP-factor < 1 × 10^5^ show acceptable 5-year OS rates and could be treated by hepatectomy.

## Background

Hepatectomy for hepatocellular carcinoma (HCC) has the highest local controllability of all local treatments for this disease and achieves a good survival rate [1, 2]. However, according to the Barcelona Clinic Liver Cancer (BCLC) staging classification [3], hepatic resection is contraindicated in intermediate-stage patients (stage B HCC) and only transarterial chemoembolization (TACE) is indicated [3]. In contrast, some patients with stage B HCC, according to the BCLC staging classification, are indicated for hepatectomy according to a Japanese evidence-based treatment algorithm [4] and a consensus-based treatment algorithm for HCC proposed by the Japan Society of Hepatology (JSH algorithm) [5], with acceptable prognoses [1]. Thus, the indication for hepatectomy differs among several staging systems.

Moreover, stage B HCC in the BCLC staging classification shows considerable heterogeneity because this stage includes tumor-related factors, namely, up to three tumors where at least one is >3 cm in diameter, more than three tumors of any size, or single tumors exceeding 5 cm in diameter. These classifications of the BCLC staging classification, JSH algorithm, and Japanese evidence-based treatment algorithm rely on tumor morphologic factors and not biomarkers such as alpha-fetoprotein (AFP) and protein induced by vitamin K absence or antagonism factor II (PIVKA-II).

In this present study, we analyzed the survival outcomes of consecutive patients who underwent primary curative hepatectomy for HCC at our center to identify the factors related to the prognosis and recurrence of stage B HCC in the BCLC staging system and newly classify this stage according to the factors identified. Using our new classification, we sought to evaluate the indications for, and significance of, performing a hepatectomy for a stage B HCC as defined by the BCLC staging system.

## Methods

### Patients

Between May 1996 and October 2014, 776 consecutive adult patients underwent hepatectomy for HCC at our center. Patients were classified into five groups in accordance with the BCLC staging system: stage 0 (*n* = 55), stage A (*n* = 327), stage B (*n* = 297), and stage C (*n* = 97). These patients were selected according to our algorithm, which incorporates the indocyanine green retention rate at 15 min (ICGR15) and remnant liver volume to determine the operative indication [6]. TACE was preoperatively performed in 44 (14.8%) patients, percutaneous ethanol injection therapy in 2 (0.7%), radiofrequency ablation in 3 (1%). As we previously reported [7], AP-factor (AFP × PIVKA-II) was categorized into three groups according to the serum concentrations of AFP and PIVKA-II, i.e., AP1 (AFP < 200 ng/ml and PIVKA-II < 100 mAU/ml), AP2 (AFP × PIVKA-II < 10^5^), and AP3 (AFP × PIVKA-II ≥ 10^5^).

All analyses in our present study were performed in accordance with the ethical guidelines of Hokkaido University Hospital. This study was approved by the Institutional Review Board of Hokkaido University.

### Hepatectomy

Anatomical resection is defined as complete anatomical removal of lesion(s) on the basis of Couinaud’s classification (segmentectomy, sectionectomy, and hemihepatectomy or extended hemihepatectomy) in patients with sufficient functional reserve. Non-anatomical partial but complete resection was achieved in all patients. R0 resections were performed in all stage B patients, and all resection surfaces were found to be histologically free of HCC.

### HCC recurrence

For the first 2 years after hepatectomy, patients underwent follow-up evaluation every 3 months comprising liver function tests, measurements of AFP and PIVKA-II, US, and dynamic computed tomography (CT). After 2 years, routine CT was performed once every 4 months. If recurrence was suspected, CT and magnetic resonance imaging (MRI) were performed, as well as CT angiography and bone scintigraphy, if necessary. This enabled precise determination of the site, number, size, and extent of invasiveness of the recurrent lesions.

### Statistical analysis

The overall survival (OS) and disease-free survival (DFS) rates were calculated using the Kaplan–Meier method and compared between groups using the log-rank test. Potential prognostic factors were identified by univariate analysis using the log-rank test. Independent prognostic factors were evaluated using a Cox proportional hazards regression model. In this study, *p* < 0.05 was considered significant. Statistical analyses were performed using JMP (version 12 for Windows; SAS Institute, Cary, NC).

## Results

### Demographics of stage B patients

The mean age of the patients with HCC Stage B was 63.7 years (range, 18–89 years). Of the 297 patients with Stage B disease, 253 (85.2%) were male, 105 (35.4%) were hepatitis B virus surface antigen-positive, and 87 (29.3%) were hepatitis C virus antibody-positive. In addition, 290 (97.6%) were categorized as Child-Pugh class A and 67 (22.6%) was diagnosed as liver cirrhosis pathologically. The tumors of 33 (11.1%) patients was limited to one segment, those of 83 (27.9%) to one section, those of 131 (44.1%) in two sections, those of 35 (11.8%) to three sections, and those of 15 (5.1%) involving more than three sections. Non-anatomical partial resection was performed in 56 (18.9%) patients. Anatomical resection of segmentectomy, sectionectomy, and hemihepatectomy/extended hemihepatectomy were performed in 27 (9%), 64 (21.5%), and 150 (50.5%) patients, respectively. The patients were followed up for a median of 98.3 months (range, 6.8–226.8 months). Twenty one patients (0.098%) were lost to follow-up for more than 5 years from the last confirmed date.

### Sites of HCC recurrence and treatment

Of the 297 stage B patients analyzed, 211 (71.0%) showed recurrence. Of the 94 recurrent cases from group A, 59 (62.8%) had recurrence only in the liver and 35 at extrahepatic sites (including or excluding the liver). Of the 99 recurrent cases in group B, 47 (47.5%) had recurrence only in the liver and 52 at extrahepatic sites. Of the 18 recurrent cases of group C, 9 (50.0%) had recurrence only in the liver and 9 in extrahepatic sites. Although there were no significant differences among the groups, recurrence in group A patients tended to occur only in the liver, whereas it tended to occur in extrahepatic sites in group B and C patients (*p* = 0.095).

We treated HCC recurrence by repeated hepatectomy (*n* = 21), transcatheter arterial chemoembolization (*n* = 135), local ablation (*n* = 12), and resection of extrahepatic metastasis (*n* = 19).

### Survival outcomes and causes of death

The 5-year OS rates for the stage 0, A, B, and C HCC cases were 85.8, 76.3, 54.3, and 19.0%, respectively. The corresponding 5-year DFS rates were 47.1, 38.2, 21.9, and 10.2%, respectively. There were 130 deaths among our 297 stage B patients (43.8%). The causes of death in these cases were HCC recurrence (*n* = 106; 81.5%), liver failure (*n* = 7; 5.4%), and other causes (*n* = 17; 16.1%). The operative mortality rate was 0.34% (1/297).

### Univariate and multivariate analyses of survival rates

By univariate analysis, significant risk factors for the reduced survival of stage B patients included serum albumin level, AFP, PIVKA-II, AP-factor, anatomical resection, blood loss, tumor number, microscopic portal vein invasion, and a noncancerous liver (Table 1). The significant risk factors for recurrence were serum albumin level, PIVKA-II, AP-factor, a higher tumor number, microscopic portal vein invasion, and microscopic hepatic vein invasion (Table 1). Multivariate analysis by the Cox proportional hazard model was then performed using these significant factors to further clarify the risk factors for lower survival and HCC recurrence. The significant factors identified by univariate analysis for stage B HCC patient survival were included in multivariate analysis which showed that the lower serum albumin level (*p* = 0.0024), nonanatomical resection (*p* = 0.0132), AP-factor (AP2 vs AP3, *p* = 0.0023), tumor number (1 vs ≥4, *p* = 0.0001; 2 or 3 vs ≥4, *p* = 0.0011) and microscopic portal vein invasion (vp0 vs vp2, *p* = 0.0001; vp1 vs vp2, *p* = 0.0133) were independent risk factors for survival (Table 2). The significant factors identified by univariate analysis for stage B HCC patient recurrence were included in multivariate analysis which showed that the lower serum albumin level (*p* = 0.026), PIVKA-II (100–1000 vs >1000, *p* = 0.038; 100–1000 vs ≤100, *p* = 0.002), AP-factor (AP1 vs AP2, *p* = 0.0036; AP2 vs AP3, *p* = 0.001), tumor number (1 vs 2 or 3, *p* = 0.037; 1 vs ≥4, *p* < 0.001) and microscopic portal vein invasion (vp0 vs vp2, *p* = 0.006) (Table 3).

**Table 1:** Univariate analysis of variables predictive (clinical and tumor associated factors) for stage B HCC patient survival and recurrence

Variables			*p* value	*p* value
		*n*	Survival	Recurrence
Sex	Male	253	0.9880	0.1503
	Female	44		
Age	<60	117	0.9306	0.4414
	≥60	180		
HBV	Negative	191	0.0516	0.2386
	Positive	105		
HCV	Negative	210	0.7523	0.2126
	Positive	87		
Albumin (g/dl)	<4	139	0.0006	0.0439
	≥4	158		
Total bilirubin (mg/dl)	<0.8	160	0.2827	0.4807
	≥0.8	137		
ICGR15 (%)	<15	178	0.3471	0.1136
	≥15	119		
Tumor number	1	145	<0.0001 ^*^1	<0.0001 ^*^2
	2or3	100		
	≥4	52		
Tumor size (cm)	≤3	8	0.9998	0.7932
	3–5	54		
	≥5	235		
Anatomical resection	Yes	241	0.0182	0.2306
	No	56		
Blood loss (ml)	<1100	239	0.0027	0.1937
	≥1100	58		
AFP (ng/ml)	≤200	201	0.0028 ^*^3	0.1276
	200–1000	26		
	>1000	70		
PIVKA-II (mAU/ml)	≤100	82	0.0354 ^*^4	0.0048 ^*^5
	100–1000	75		
	>1000	139		
AP-factor (AFP*PIVKA-II)	AP1	65	<0.0001 ^*^6	0.0009 ^*^7
	AP2	99		
	AP3	133		
Differentiation	Well	19	0.2092	0.1655
	Moderate	160		
	Poor	115		
	Unknown	19		
Microscopic portal vein invasion	vp0	205	0.0003 ^*^8	0.0103 ^*^9
	vp1	62		
	vp2	28		
	Unknown	1		
Microscopic hepatic vein invasion	vv0	271	0.01463 ^*^10	0.0262^*^11
	vv1	25		
	Unknown	1		
Non-cancerous liver	Cirrhosis	67	0.1328	0.1772
	Non-cirrhosis	230		

HbsAg, hepatitis B virus s antigen; HCV, anti-hepatitis C virus antibody; AFP, alpha-fetoprotein; PIVKA-II, protein induced by vitamin K absence or antagonism factor II; ICGR15, indocyanine green retention rate at 15 min; AP-factor, a product of the serum levels of AFP and PIVKA-II. HCC patients were classified into three groups: AP1 (AFP < 200 ng/ml and PIVKA-II < 100 mAU/ml), AP2 (AFP × PIVKA-II < 10^5^), and AP3 (AFP × PIVKA-II ≥ 10^5^)

vp0: no tumor thrombus in the portal vein

vp1: tumor thrombus distal to the second branches of the portal vein

vp2: tumor thrombus in the second branches of the portal vein

vv0: no tumor thrombus in the hepatic vein

vv1: tumor thrombus in a branch of the hepatic vein

When the subgroups were more than three, *p*-value in Table 1 was showed a significant difference as a group. *P*-value between the subgroups was showed with asterisk

^*^1: tumor number 1 vs ≥4 (*p* < 0.0001), tumor number 2or3 vs ≥4 (*p* = 0.0003)

^*^2: tumor number 1 vs 2or3 (*p* = 0.0047), tumor number 1 vs ≥4 (*p* < 0.0001), tumor number 2or3 vs ≥4 (*p* = 0.0122)

^*^3: AFP ≤ 200 vs 200–1000 (*p* = 0.0072), ≤200 vs >1000 (*p* = 0.0009)

^*^4: PIVKA-II ≤100 vs >1000 (*p* = 0.0043)

^*^5: PIVKA-II ≤100 vs 100–1000 (*p* = 0.0009), ≤100 vs >1000 (*p* = 0.0224)

^*^6: AP-1 vs Ap-3 (*p* = 0.0001), AP-2 vs Ap-3 (*p* < 0.0001)

^*^7: AP-1 vs Ap-3 (*p* = 0.0012), AP-2 vs Ap-3 (*p* = 0.0074)

^*^8: vp0 vs vp1 (*p* = 0.0005), vp0 vs vp2 (*p* < 0.0001)

^*^9: vp0 vs vp2 (*p* = 0.0024)

^*^10: vv0 vs vv1 (*p* = 0.0123)

^*^11: vv0 vs vv1 (*p* = 0.0181)

**Table 2:** Multivariate analysis of variables predictive for stage B HCC patient survival

Survival	*p* value	Risk ratio	95% confidence interval	
Albumin (g/dl), <4 vs ≥4	0.0024	1.837	1.241	2.728
Anatomical vs nonanatomical	0.0132	0.509	0.304	0.866
AP-factor, AP2 vs AP3	0.0023	0.416	0.234	0.732
Tumor number, 1 vs 4over	0.0001	0.380	0.229	0.615
2,3 vs ≥4	0.0011	0.414	0.248	0.699
Microscopic portal vein invasion, vp0 vs vp2	0.0001	0.302	0.174	0.545
vp1 vs vp2	0.0133	0.435	0.231	0.837

PIVKA-II, protein induced by vitamin K absence or antagonism factor II; AP-factor, a product of the serum levels of AFP and PIVKA-II

vp0: no tumor thrombus in the portal vein

vp1: tumor thrombus distal to the second branches of the portal vein

vp2: tumor thrombus in the second branches of the portal vein

**Table 3:** Multivariate analysis of variables predictive for stage B HCC patient recurrence

Recurrence	*p* value	Risk ratio	95% confidence interval	
Albumin (g/dl), <4 vs ≥4	0.026	1.382	1.040	1.834
PIVKA-II (mAU/ml), ≤100 vs >1000	0.437	0.537	0.266	0.983
100–1000 vs >1000	0.038	1.479	1.023	2.127
100–1000 vs ≤100	0.002	2.755	1.447	5.717
AP-factor, AP1 vs AP2	0.036	2.102	1.049	4.548
AP2 vs AP3	0.001	0.562	0.395	0.795
Tumor number, 1 vs 2,3	0.037	0.709	0.514	0.979
1 vs ≥4	<0.001	0.485	0.326	0.739
Microscopic portal vein invasion, vp0 vs vp2	0.006	0.488	0.310	0.805

PIVKA-II, protein induced by vitamin K absence or antagonism factor II; AP-factor, a product of the serum levels of AFP and PIVKA-II

vp0: no tumor thrombus in the portal vein

vp1: tumor thrombus distal to the second branches of the portal vein

vp2: tumor thrombus in the second branches of the portal vein

### Overall and disease-free survival outcomes categorized by tumor number and AP-factor

**Fig. 1:**
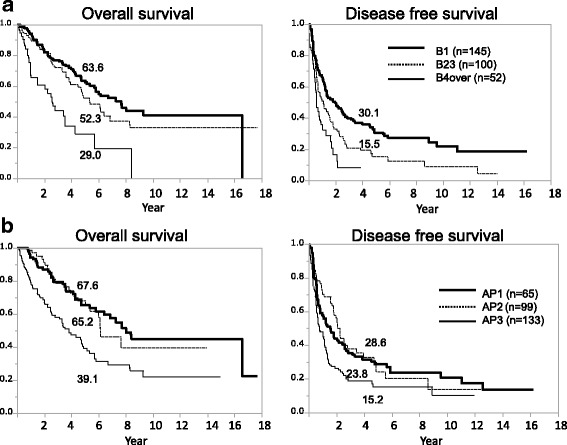
Overall survival curves according to tumor number and AP-factor. **a** The 5-year overall survival rates of B1, B23, and B4over were 63.6, 52.3, and 29.0%, respectively. There was a significant difference between each of these groups (*p* < 0.01), except for B1 and B23. The 5-year disease-free survival rates of B1 and B23 were 30.1 and 15.5%, respectively (*p* < 0.01). **b** The 5-year overall survival rates of AP1, AP2, and AP3 were 67.6, 65.2, and 39.1%, respectively. There was a significant difference between AP1 and AP3 (*p* < 0.01) and between AP2 and AP3 (*p* < 0.01). The 5-year disease-free survival rates of AP1, AP2, and AP3 were 23.8, 28.6, and 15.2%, respectively. There was a significant difference between AP1 and AP3 (*p* < 0.01) and between AP2 and AP3 (*p* < 0.01)

The study patients with stage B HCC were classified into three groups by tumor number (B1, 1; B23, 2 or 3; B4over: ≥4) and into three groups stratified by AP-factor (AP1, AP2, and AP3). This grouping was done because the HCC tumor number and AP-factor were found to be risk factors for poorer survival and recurrence by multivariate analysis and these are tumor-related factors that can be evaluated preoperatively. The 5-year OS rates for B1, B23, and B4over cases were 63.6, 52.3, and 29.0%, respectively. There was a significant difference between each of these groups (*p* < 0.01) except between B1 and B23. There was no difference in OS between the tumor number 2 and 3 (*p* = 0.8224). The 5-year DFS rates for B1 and B23 were 30.1 and 15.5%, respectively (*p* < 0.01) (Fig. 1a). The 5-year OS rates for the AP1, AP2, and AP3 cases were 67.6, 65.2, and 39.1%, respectively. There was a significant difference between AP1 and AP3 (*p* < 0.01) and between AP2 and AP3 (*p* < 0.01). The 5-year DFS rates for the AP1, AP2, and AP3 patients were 23.8, 28.6, and 15.2%, respectively. There were also significant differences between AP1 and AP3 (*p* < 0.01) and between AP2 and AP3 (*p* < 0.01; Fig. 1b).

### Stage B patients stratified by tumor number and AP-factor

**Fig. 2:**
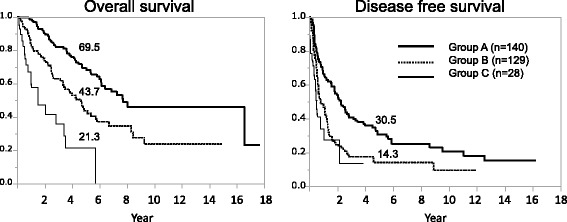
Overall survival curves for groups A, B and C. Overall survival curves for groups A-C with 5-year patient survival rates of 69.5, 43.7, and 21.3%, respectively. There was a significant difference between each of these groups (*p* < 0.01). The 5-year disease-free survival rates of group A and group B were 30.5 and 14.3%, respectively (*p* < 0.01)

Stage B patients were further categorized in nine ways by combining tumor number and AP-factor which are tumor-related factors that can be evaluated preoperatively. The 5-year OS rates of these nine categories ranged from 21.3 to 75.6% (Table 4). Stratified by the 5-year OS rate, stage B HCC patients were classified into three subgroups (A-C). Group A included patients with a tumor number ≤ 3 and AP-factor < 1 × 10^5^, group B included patients with tumor number ≤ 3 and AP-factor > 1 × 10^5^ or tumor number ≥ 4 and AP-factor < 1 × 10^5^, and group C incorporated patients with tumor number ≥ 4 and AP-factor > 1 × 10^5^. The 5-year PS rates of groups A-C were 69.5, 43.7, and 21.3%, respectively. There were significant differences between each of these groups (*p* < 0.01). The 5-year DFS of groups A and B were 30.5 and 14.3%, respectively (*p* < 0.01; Fig. 2).

**Table 4:** Categorization of patients into nine categories by tumor number and AP-factor and subclassification of BCLC stage B HCC

	AP-1	AP-2	AP-3	(*n*)
B1	75.6%	73.7%	45.3%	(145)
	(35)	(53)	(57)	
B23	57.1%	65.6%	43.0%	(100)
	(25)	(27)	(48)	
B4over	60.0%	34.7%	21.3%	(52)
	(5)	(19)	(28)	
(n)	(65)	(99)	(133)	(297)

AP-factor, a product of the serum levels of AFP and PIVKA-II. HCC patients were classified into three groups: AP1 (AFP < 200 ng/ml and PIVKA-II < 100 mAU/ml), AP2 (AFP × PIVKA-II < 10^5^), and AP3 (AFP × PIVKA-II ≥ 10^5^). B1, B23, and B4over were classified by tumor numbers 1, 2 or 3, and ≥4, respectively. Group A comprises B1 or B23 and AP-1 or AP-2. Group B comprises B1 or B23 and AP-3, or B4over and AP-1 or AP-2. Group C comprises B4over and AP-3

## Discussion

In our present study, univariate and multivariate analysis was used to identify risk factors in patients with BCLC stage B HCC. Of the risk factors identified for both survival and recurrence, tumor number and AP-factor can be determined preoperatively. Our stage B patients were categorized into nine groupings according to these two risk factors. Based on the 5-year OS rates among these nine categories, stage B patients were classified into three subgroups (A-C) with 5-year OS rates of 69.5, 43.7, and 21.3%, respectively. Even in stage B HCC cases, the 5-year OS rates of some patients with a tumor number ≤ 3 and/or AP-factor < 1 × 10^5^ ranged from 43.7 to 69.5%. These patients could therefore be treated with hepatectomy.

The main limit of BCLC staging is the considerable prognostic heterogeneity within each stage. Stage B HCC under this system is composed of a heterogeneous population because the classifications are done using tumor status, liver function, and physical status [3]. In recent years, Bolondi et al. [8] and Kudo et al. [9] proposed modified models of the BCLC staging system for prognostic prediction in HCC patients. However, because these suggested subclassifications were conducted to decide on the best TACE treatment strategy, they were limited in helping clinicians with hepatectomy-related treatment decisions. Wada et al. [10] reported on the selection criteria for hepatic resection in BCLC stage B HCC. However, their subclassification relied on tumor number and size and not on tumor markers. The indications for a large HCC should also be reconsidered due to reports on the biological malignancy of solitary large HCC. It has been proposed that solitary large HCC should be considered a specific subtype that is less malignant than nodular HCC, because the expression levels of some human genes closely related to invasion and metastasis are significantly lower in these lesions [11]. It has also been reported that after resection of solitary large HCC, the clinical outcomes are similar to those of small HCC but are significantly better than those in patients with multiple HCC [12]. This study and the report by Torzilli G [13] included large solitary HCC (above 5 cm in diameter) as BCLC B HCC. Therefore, the classification of solitary tumors above 5 cm should be characterized as BCLC B by the staging system.

Given the aforementioned issues, a reliable biomarker to determine the biological malignancy of HCC must be included in the current staging system. AFP and PIVKA-II have been used previously as useful tumor markers of HCC and are associated with a poor prognosis after hepatectomy [14, 15]. AFP is related to tumor differentiation [16], whereas PIVKA-II is related to vascular invasion [17, 18]. Although the latter is the most influential factor for recurrence and survival in patients undergoing hepatectomy [19], the oncological implications of AFP and PIVKA-II remain unclear. We previously established the AP-factor, a product of the serum levels of AFP and PIVKA-II, as a critical marker for preoperatively predicting tumor malignancy [7]. In the present study, tumor number and AP-factor were identified as the tumor-related risk factors for survival and recurrence by multivariate analysis that could be evaluated preoperatively. We could clearly categorize our BCLC stage B patients into three subgroups by tumor number and AP-factor; the 5-year OS rates of these subgroups (A-C) were 69.5, 43.7, and 21.3%, respectively. Notably, Yamakado et al. previously reported a 5-year overall survival rate of 23.7% in BCLC B patients treated with TACE [20]. Hence, the operative success of groups A and B was acceptable. Moreover, Kim et al. reported that after propensity score matching in BCLC B patients, the 1-, 2-, 3-, and 5-year overall survival rates in the resection group were 90, 88, 75, and 63%, compared with 79, 48, 35, and 22% in the no-surgery group whom 94% had TACE as the first treatment (*P* < 0·001) [21].

Because approximately 90% of patients with HCC also have hepatitis B and/or hepatitis C, and thus have chronic hepatitis or cirrhosis [22], the liver functional reserve was decreased in almost all of our current study patients. Liver resection in cirrhotic patients is thus associated with high mortality rates (between 8.9 and 19.6%) [23]. Conversely, recent advances in surgical techniques and pre- and postoperative care, including the decision criteria for hepatectomy and indications for liver resection, have been applied to extended hepatectomy [6, 24]. These preoperative evaluations allow a major hepatectomy to be more safely performed, although operative mortality is never completely avoided, even in donor hepatectomy for living donor liver transplantation [25]. In our present study, the operative mortality rates were as low as 0.39%. Moreover, the large multicentric survey of Torzilli G and colleagues reported a 90-day mortality rate of 2.7% and a 5-year overall survival rate of 61% for BCLC B patients who underwent surgery [13]. Because hepatectomy is not as invasive and does not impose a large burden on patients from the point of view of operative mortality, we contend that the indications for hepatectomy could be expanded in BCLC stage B HCC cases.

Hepatectomy is associated with a high risk of recurrence. Thus, it has been proposed that salvage transplantation be considered after hepatectomy in HCC patients with preserved liver function [26, 27]. Recurrence patterns after curative hepatectomy have been analyzed previously in patients classified according to the Milan criteria [28]. In that study, about 30% of the patients whose lesions exceeded the Milan criteria did not develop HCC recurrence. About half of the studies patients with HCC recurrence met the Milan criteria, representing about 30% of those who exceeded the Milan criteria. HCC that was even beyond the Milan criteria was considered an indication for salvage transplantation. This further supports our belief that hepatectomy could be indicated for HCC stage B.

## Conclusions

In conclusion, the 5-year overall survival rates of patients with tumor number ≤ 3 and/or AP-factor < 1 × 10^5^ were acceptable even in BCLC stage B HCC patients. These patients could be treated with hepatectomy. Hepatectomy could also be performed after careful consideration in HCC patients with a tumor number ≥ 4 and AP-factor > 1 × 10^5^.

## Abbreviations

AFP: Alpha-fetoprotein
AP-factor: AFP × PIVKA-II
BCLC: Barcelona Clinic Liver Cancer
CT: Computed tomography
DFS: Disease-free survival
HCC: Hepatocellular carcinoma
ICGR15: Indocyanine green retention rate at 15 min
MRI: Magnetic resonance imaging
OS: Overall survival
PIVKA-II: Protein induced by vitamin K absence or antagonism factor II
TACE: Transarterial chemoembolization
